# A Prediction Model for Prediabetes Risk in Middle-Aged and Elderly Populations: A Prospective Cohort Study in China

**DOI:** 10.1155/2021/2520806

**Published:** 2021-11-11

**Authors:** Jiahua Wu, Jiaqiang Zhou, Xueyao Yin, Yixin Chen, Xihua Lin, Zhiye Xu, Hong Li

**Affiliations:** Department of Endocrinology, Sir Run Run Shaw Hospital, Zhejiang University School of Medicine, 3 East Qingchun Road, Hangzhou 310016, China

## Abstract

**Background:**

To investigate indicators for prediabetes risk and construct a prediction model for prediabetes incidences in China.

**Methods:**

In this study, 551 adults aged 40–70 years had normal glucose tolerance (NGT) and normal hemoglobin A1c (HbA1c) levels at baseline. Baseline data including demographic information, anthropometric measurements, and metabolic profile measurements were collected. The associations between possible indicators and prediabetes were assessed by the Cox proportional-hazards model. The predictive values were evaluated by the area under the receiver operating characteristic (ROC) curve (AUC).

**Results:**

During an average of 3.35 years of follow-up, the incidence of prediabetes was found to be 19.96% (*n* = 110). In the univariate analyses, fasting plasma glucose (FPG), fasting serum insulin (FINS), 2 h plasma glucose (2hPG), HbA1c, serum uric acid (SUA), waist circumference (WC), smoking, and family history of diabetes (FHD) were found to be significantly correlated with prediabetes. In the multivariable analyses, WC (hazard ratio (HR): 1.032; 95% confidence interval (CI): 1.010, 1.053; *p* = 0.003), FHD (HR: 1.824; 95% CI: 1.250, 2.661; *p* = 0.002), HbA1c (HR: 1.825; 95% CI: 1.227, 2.714; *p* = 0.003), and FPG (HR: 2.284; 95% CI: 1.556, 3.352; *p* < 0.001) were found to be independent risk factors for prediabetes. A model that encompassed WC, FHD, HbA1c, and FPG for predicting prediabetes exhibited the largest discriminative ability (AUC: 0.702).

**Conclusions:**

WC, FHD, HbA1c, and FPG are independently correlated with the risk of prediabetes. Furthermore, the combination of these predictors enhances the predictive accuracy of prediabetes.

## 1. Introduction

Diabetes has evolved as a global health challenge. It is correlated with a high mortality rate, increased health risks, medical costs, and a poor quality of life. Prediabetes refers to the transition from normal glucose metabolism to diabetes, consisting of impaired fasting glucose (IFG) and impaired glucose tolerance (IGT) [1]. Prediabetic adults do not show any signs or symptoms of diabetes; therefore, many people are unaware that they are living with prediabetes [2]. Prediabetic individuals have an increased risk for diabetes, obesity, hypertension, dyslipidemia, cardiovascular disease, cancer, and dementia [3, 4]. Prediabetes prevention and control reduces the chances for progression to diabetes and its related complications.

In 2017, the World Health Organization documented that the global prevalence of diabetes and prediabetes has been on the rise [5]. Globally, China has the highest number of prediabetes patients, with a prevalence of 35.2% in 2017. This corresponds to approximately 357 million people [6]. A Chinese study involving adults with IGT reported that the cumulative incidence of type 2 diabetes in 6 years was 67.7% [7]. Therefore, it is important to identify indicators that efficiently predict prediabetes development. This will help monitor patients and improve intervention.

Diabetes pathogenesis involves interactions between genes and the environment. Factors influencing prediabetes include age [8, 9], educational attainment [9], marital status [9], hypertension [10], dyslipidemia [11], gestational diabetes, body mass index (BMI) [9, 12], WC [12], FHD [13], diet patterns [14], and 1-hour plasma glucose levels [15]. In Japan, a prognostic model based on six variables was built to predict the incidences of type 2 diabetes mellitus and prediabetes in a healthy population [16]. The AUC of the predictive model was found to be 0.87. Moreover, a model for screening prediabetes in Indonesian adults (AUC = 0.623), which includes age, sex, education level, FHD, smoking, physical activity, BMI, and hypertension, has been developed and verified [17]. However, a prediction model for predicting prediabetes incidences in China has not been constructed. Therefore, we aimed at investigating the potential indicators and to construct a prediction model for prediabetes incidence among middle-aged and elderly populations in urban areas of Hangzhou, China.

## 2. Methods

### 2.1. Study Participants

Participants enrolled in this cohort study were from the Caihe community of Jianggan District, Hangzhou. Participants, aged between 40 and 70 years, were recruited by community workers. Recruitment was done between January and March 2010. Follow-up was done in 2011, 2013, and 2015. The exclusion criteria were as follows: (i) those using glucocorticoids; (ii) those with cirrhosis and ascites; (iii) hyperthyroidism or hypothyroidism; (iv) cancer; (v) severe disabilities or mental diseases; (vi) pregnant and lactating women; (vii) participants with diabetes or prediabetes at baseline; (viii) those with incomplete information; (ix) lost at follow-up; and (x) new-onset diabetes before the diagnosis of prediabetes at follow-up. For all participants, there was at least one visit between 2011 and 2015. The study was approved by the ethics committee of Sir Run Run Shaw Hospital. Informed consent was provided by all participants.

### 2.2. Demographic Characteristics

Age, gender, education levels, nature of occupation, cigarette smoking habits, alcohol drinking habits, dietary patterns, regular exercise, and FHD data were obtained by a questionnaire. Participants were grouped based on cigarette smoking habits, that is, smoker, exsmoker, and nonsmoker. Moreover, they were categorized based on alcohol drinking habits, that is, drinker, exdrinker, and nondrinker. Education levels were categorized as middle school or above and below middle school. The nature of occupation before retirement was defined as manual work, physical and mental work, and mental work. Dietary patterns were divided into main meat dishes, balanced meat and vegetables, and main vegetable dishes. The FHD was defined as the presence of diabetes among first-degree relatives. Regular exercise was more than 1 day per week.

### 2.3. Assessment of Anthropometric Measurements and Metabolic Profiles

The measurement of WC, blood pressure, BMI, FPG, FINS, SUA, high-density lipoprotein cholesterol (HDL-C), triglyceride (TG) levels, and 2hPG levels was based on the previous article [18].

### 2.4. Definition

The definition of hypertension was systolic blood pressure (SBP) ≥140 mmHg and/or diastolic blood pressure (DBP) ≥90 mmHg and/or diagnosed as hypertension by a physician previously. The definition of diabetes was FPG ≥7.0 mmol/L, 2hPG ≥11.1 mmol/L, or previously diagnosed as diabetes. The definition of prediabetes was 7.0 mmol/L > FPG ≥ 6.1 mmol/L, 11.1 mmol/L > 2hPG ≥ 7.8 mmol/L, or previously diagnosed as prediabetes.

### 2.5. Statistical Analysis

All analyses were performed with R 3.5.0 and SPSS 26.0. Continuous variables were shown as means ± standard deviations, medians (interquartile ranges), or frequencies and percentages. Continuous variables with a skewed distribution were transformed by natural logarithm transformation before analysis. Comparison between 2 groups was conducted by independent-samples *t*-test for continuous variables, while *χ*^2^ tests were used for categorical variables. The multivariable Cox model was performed to estimate the correlations between indicators and prediabetes incidences. Clinically relevant baseline variables or variables with *p* < 0.2 upon univariate analysis were entered into the multivariate analysis. Variables were entered into the multivariate Cox proportional-hazards model one by one. They were kept in the final models, which were added to this model, changing the matched hazard ratio by at least 10 percent or if the *p* value by itself made sense. The variance inflation factor of the variables included in the model was examined to address collinearity. No evidence of collinearity was noted in the model, given the variance inflation factor of <5. The ROC was applied to compare the predictive accuracy of various models. Differences between AUC were determined using DeLong's test. Additionally, integrated discrimination improvement (IDI), net reclassification index (NRI), and Akaike's information criterion (AIC) were calculated to evaluate the predictive values of different models. *p* ≤ 0.05 was considered statistically significant.

## 3. Results

### 3.1. Basic Characteristics of the Study Participants

**Figure 1:**
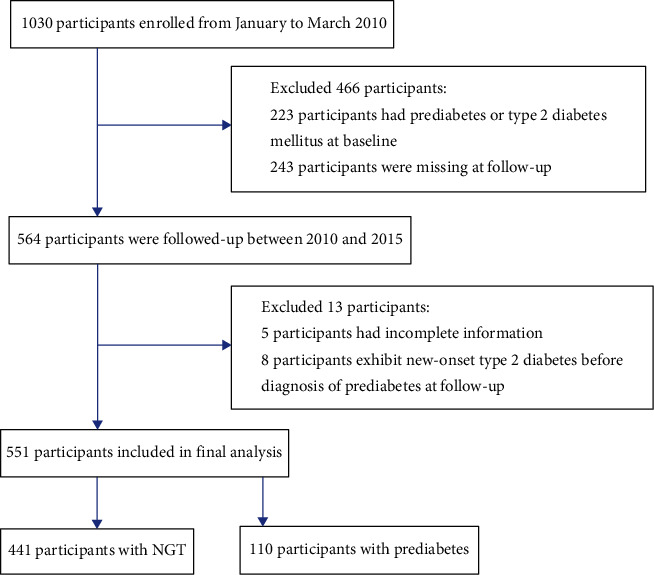
Schematic presentation for participant selection.

A total of 1,030 participants were initially enrolled from January to March 2010. Among them, 223 had prediabetes or type 2 diabetes at baseline, 243 were lost at follow-up, while 5 participants had incomplete data. Eight participants were excluded because they were diagnosed as type 2 diabetes at follow-up. Finally, 551 eligible participants were enrolled (Figure 1). During an average of 3.35 years of follow-up, 110 of the 551 participants without dysglycemia at baseline developed prediabetes (incidence: 19.96%). Demographic, clinical, and biological characteristics of 551 participants are shown in Table 1. Average age at baseline was 53.23 ± 6.62 years in the NGT group and 54.42 ± 7.02 years in the prediabetes group. Participants with prediabetes outcomes exhibited significantly elevated WC, BMI, FPG, 2hPG, HbA1c, FINS, and SUA levels as well as significantly suppressed HDL-C level at baseline. Moreover, at baseline, proportion of male, prevalence of hypertension, smoking, and FHD were significantly higher in participants with prediabetes. At baseline, there were no significant differences in age, TG, alcohol drinking, dietary patterns, nature of occupation, education level, and regular exercise.

### 3.2. Associations between Possible Indicators and the Risk of Prediabetes

In the univariate analyses (Additional file 1: Table S1), FPG, FINS, 2hPG, HbA1c, SUA, WC, smoking, and FHD were found to be significantly correlated with prediabetes. The FPG was the strongest predictor for prediabetes. In the multivariate model, 16 variables (age, gender, hypertension, smoking, drinking, FHD, regular exercise, WC, BMI, FPG, FINS, 2hPG, HbA1c, SUA, HDL-C, and TG) were selected into the model for screening. The final prognostic models are shown in Table 2. Given the relationship between WC and glucose metabolism, WC was set as the base model (model 1), which was easy to be clinically obtained. Model 2 additionally included FHD. The variables in model 2 plus HbA1c were selected for model 3. Model 4 additionally included FPG. Multivariate Cox proportional-hazard analyses showed that WC (HR: 1.032; 95% CI: 1.010, 1.053; *p* = 0.003), FHD (HR: 1.824; 95% CI: 1.250, 2.661; *p* = 0.002), HbA1c (HR: 1.825; 95% CI: 1.227, 2.714; *p* = 0.003), and FPG (HR: 2.284; 95% CI: 1.556, 3.352; *p* < 0.001) were independent risk factors of prediabetes.

### 3.3. Predictive Values of FPG, HbA1c, WC, and FHD for Prediabetes

**Figure 2:**
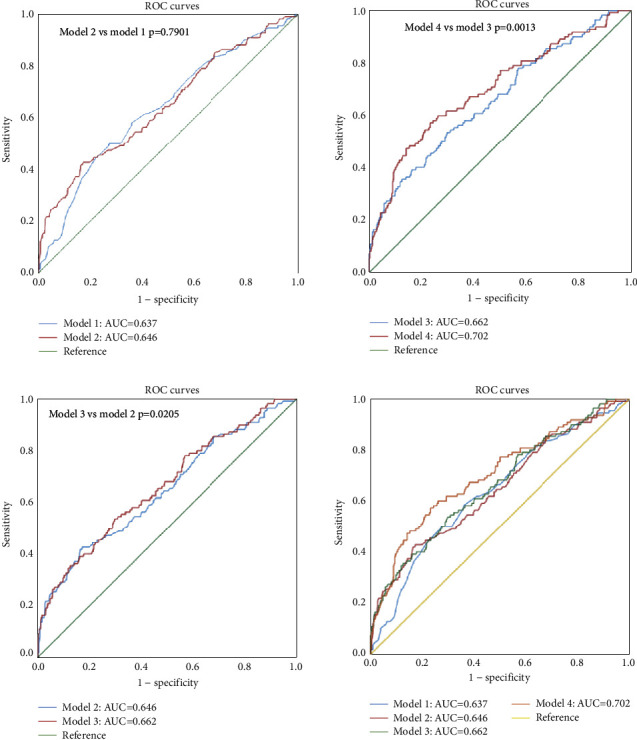
Area under the receiver operating characteristic curves for predicting prediabetes. Model 1: incorporating WC. Model 2: variables in model 1 and FHD. Model 3: variables in model 2 and HbA1c. Model 4: variables in model 3 and FPG. FHD: family history of diabetes; FPG: fasting plasma glucose; HbA1c: hemoglobin A1c; WC: waist circumference.

The predictive accuracy of the resultant models for prediabetes was evaluated by ROC curve analysis (Figure 2). The established independent risk factors for prediabetes were used to construct the predictive model. The AUC was 0.637 when WC was used alone (model 1). The addition of FHD (model 2) in model 1 did not significantly increase the AUC (model 2 versus model 1: DeLong's test, *p*=0.7901). However, a significant increase in AUC was observed when HbA1c and FPG were further added (model 3 versus model 2: DeLong's test, *p*=0.0205; model 4 versus model 3: DeLong's test, *p*=0.0013), with AUC eventually increasing to 0.702 (model 4). Discriminative abilities of the resultant models were confirmed using the NRI, IDI, and AIC measures (Table 3). Model 4 exhibited the smallest AIC measure and significantly enhanced risk reclassification and discrimination (NRI, 30.4%; 95% CI: 0–38.9%; IDI, 10.4%; 95% CI: 1.7–21.5%) when compared with model 1.

## 4. Discussion

In this study, we found that FPG, HbA1c, WC, and FHD are independently associated with the development of prediabetes among middle-aged and elderly adults in China. Moreover, the potential of the developed prognostic model for screening the individual risk of developing prediabetes was high.

People with visceral obesity are more likely to develop prediabetes [19]. Glucose utilization in the peripheral tissues and the liver is affected by changes in tissue proportions and increase of free fatty acids. This causes gluconeogenesis, which results in insulin resistance, thereby increasing the risk of prediabetes [20]. WC is a simple and commonly used marker for visceral obesity and is correlated with a higher risk of prediabetes among Chinese and Iranian adults [12]. Our study confirmed this finding. The WC was independently correlated with a 1.032-fold (95% CI: 1.010–1.053) increased risk of prediabetes. Despite the power of WC for predicting prediabetes, its predictive value was not strong enough as an independent predictor in the clinical setup. Other risk factors should be considered to improve its ability as a predictive tool for prediabetes.

FHD is a predictor for prediabetes development. A multicenter study found that FHD was significantly correlated with prediabetes development [21]. This relationship remained significant when adjusted for gender, age, and BMI. Moreover, FHD in a first-degree relative was associated with IFG among children and adolescents [22]. In this study, FHD was independently correlated with a 1.824-fold (95% CI: 1.250–2.661) increased risk of prediabetes. The results are in line with the reported familial clustering risk and may be related to genetics and family environment [23].

Besides WC and FHD, FPG was correlated with a higher risk for prediabetes in our study. FPG was independently correlated with a 2.284-fold (95% CI: 1.556–3.352) increased risk of prediabetes. This outcome has been confirmed in other studies. An increase in FPG within the normal range is correlated with increased incidences of IGT and IFG [24]. Elevated FPG (within the normal range) is a predictor for prediabetes and diabetes development during youth, middle, and old ages [25]. These studies confirm that an increase in FPG is potentially useful in identifying healthy people who are at risk of developing prediabetes. Furthermore, we found that HbA1c is correlated with a higher risk for prediabetes. This is the first work to be performed in China to determine whether HbA1c is beneficial in risk prediction of prediabetes. Interestingly, HbA1c was independently correlated with a 1.825-fold (95% CI: 1.227–2.714) increased risk of prediabetes.

Most of the domestic studies have used a single index to predict prediabetes. In a 2020 study, it was shown that the triglyceride-glucose index (TyG index) was considered as a potential predictor for identifying high-risk individuals with prediabetes. The AUC for the TyG index in predicting prediabetes was found to be 0.60 [11]. The Chinese visceral adiposity index (CVAI) is considered as a better indicator for predicting prediabetes than WC in Chinese adults. The AUC for the CVAI was found to be 0.64 in women and 0.57 in men [12]. Visceral adiposity index (VAI), waist-height ratio (WHtR), and WC were considered as independent predictors for prediabetes in women over 40 years. The AUC of VAI, WHtR, and WC for predicting prediabetes was 0.625, 0.602, and 0.598, respectively [26]. All the aforementioned predictors were not independently strong enough to predict prediabetes in the clinical setup (AUC < 0.70). A combination of different predictors enhances the predictive accuracy for prediabetes. In this study, multivariate Cox proportional-hazard analyses revealed that WC, FHD, HbA1c, and FPG were independent risk factors of prediabetes. A combination of FPG, HbA1c, WC, and FHD was input in model 4 to predict prediabetes. ROC curve analysis was finally conducted to evaluate the predictive ability of the model for prediabetes (AUC = 0.702; Figure 2). AIC, NRI, and IDI were used to compare the prediction models. They exhibited better predictive values of FPG, HbA1c, WC, and FHD for prediabetes (Table 3). This is the first study to construct a multivariate prediction model, which included demographic characteristics, anthropometric measurements, and metabolic profiles for Chinese adults.

The functions of islets gradually decline with age, including weakened insulin action, leading to lipid and glucose metabolism disorder. Prediabetes incidences are correlated with age [27]. Gender has also been reported to be correlated with prediabetes incidences. For instance, the prevalence of prediabetes was significantly higher in males than in females [28]. However, it was reported that gender and age were not correlated with prediabetes in another study [29]. This is in line with our study, potentially due to the sample size and differences in the districts of participants. This warrants further investigations.

This work has some limitations. First, the study population is from a single community in southern China, with limited representation. Therefore, the true effect of this model should be further investigated and verified in different populations. Second, correlations stratified by age and gender were unexplored because of sample size limitations. Third, although many people were lost to follow-up, no significant statistical differences were noted in basic characteristics for the 243 lost and 551 follow-up participants. Therefore, the 551 followed up participants may validly represent the entire study cohort. Despite its limitations, the model can still be used, given that few models have been developed to predict prediabetes in the Chinese population.

## 5. Conclusions

FPG, HbA1c, WC, and FHD are independently correlated with the increased risk of prediabetes. A combination of these predictors enhances the predictive accuracy for prediabetes. We developed a multivariate prognostic model to predict the risk of prediabetes development. This may help in identifying high-risk populations and to develop preventive strategies.

## Figures and Tables

**Table 1:** Basic characteristics of study participants categorized by glucose metabolism.

Characteristic	NGT (*n* = 441)	Prediabetes (*n* = 110)	*p* value
Age, years	53.23 ± 6.62	54.42 ± 7.02	0.093
Gender, men (%)	149 (33.80)	52 (47.30)	0.009
BMI, kg/m^2^	23.01 ± 2.67	23.77 ± 2.73	0.007
WC, cm	76.47 ± 8.40	80.56 ± 8.85	<0.001
FPG, mmol/L	4.70 ± 0.46	4.98 ± 0.58	<0.001
FINS, uIU/mL	5.73 ± 4.31	7.54 ± 6.41	<0.001
2hPG, mmol/L	5.03 ± 1.14	5.52 ± 1.36	<0.001
HbA1c, mmol/mol	36.32 ± 4.57	38.45 ± 4.57	<0.001
HbA1c (%)	5.47 ± 0.42	5.67 ± 0.42	<0.001
SUA, *μ*mol/L	272.77 ± 84.21	299.83 ± 81.56	0.003
^a^TG, mmol/L	1.23 (0.89–1.66)	1.44 (0.90–1.93)	0.098
HDL-C, mmol/L	1.50 ± 0.36	1.41 ± 0.37	0.015
Hypertension, *n* (%)	94 (21.30)	34 (30.90)	0.033
Family history of diabetes, *n* (%)	108 (24.50)	48 (43.60)	<0.001
Regular exercise, *n* (%)	271 (61.50)	64 (58.20)	0.530
Smoking, *n* (%)			0.003
Current smokers	92 (20.90)	26 (23.60)	
Former smokers	17 (3.90)	13 (11.80)	
Never smokers	332 (75.30)	71 (64.50)	
Alcohol drinking, *n* (%)			0.195
Current drinkers	85 (19.30)	26 (23.60)	
Former drinkers	82 (18.60)	26 (23.60)	
Never drinkers	274 (62.10)	58 (52.70)	
Dietary patterns, *n* (%)			0.789
Main meat dishes	30 (6.80)	8 (7.30)	
Balanced meat and vegetables	319 (72.30)	76 (69.10)	
Main vegetable dishes	92 (20.90)	26 (23.60)	
Nature of occupation, *n* (%)			0.240
Manual work	111 (25.20)	23 (20.90)	
Physical and mental work	183 (41.50)	41 (37.30)	
Mental work	147 (33.30)	46 (41.80)	
Education level, *n* (%)			0.914
Middle school or below	211 (47.80)	52 (47.30)	
High school or above	230 (52.20)	58 (52.70)	

Values are means ± standard deviations, *n* (%), or medians (interquartile ranges). ^a^Natural logarithm transformed before analysis. BMI: body mass index; WC: waist circumference; FPG: fasting plasma glucose; FINS: fasting serum insulin; HbA1c: hemoglobin A1c; HDL-C: high-density lipoprotein cholesterol; NGT: normal glucose tolerance; SUA: serum uric acid; TG: triglycerides; 2hPG: 2 h plasma glucose.

**Table 2:** Multivariable Cox proportional-hazards regression models for predicting prediabetes.

Variable	Model 1		Model 2		Model 3		Model 4	
	HR (95% CI)	*p* value	HR (95% CI)	*p* value	HR (95% CI)	*p* value	HR (95% CI)	*p* value
WC	1.039 (1.018, 1.061)	<0.001	1.039 (1.018, 1.060)	<0.001	1.039 (1.018, 1.061)	<0.001	1.032 (1.010, 1.053)	0.003
FHD	—	—	1.924 (1.320, 2.805)	0.001	1.844 (1.262, 2.693)	0.002	1.824 (1.250, 2.661)	0.002
HbA1c	—	—	—	—	2.028 (1.382, 2.976)	<0.001	1.825 (1.227, 2.714)	0.003
FPG	—	—	—	—	—	—	2.284 (1.556, 3.352)	<0.001

Model 1: incorporating WC; model 2: variables in model 1 and FHD; model 3: variables in model 2 and HbA1c; model 4: variables in model 3 and FPG. CI, confidence intervals; FHD, family history of diabetes; FPG, fasting plasma glucose; HbA1c, hemoglobin A1c; HR, hazard ratios; WC, waist circumference.

**Table 3:** Performance of models in prediabetes prediction.

Model	AIC	NRI (95% CI)	*p* value	IDI (95% CI)	*p* value
Model 1	1275.641	—	—	—	—
Model 2	1266.549	9.1% (−7.3, 22.8)	0.348	1.4% (−0.7, 5.7)	0.279
Model 3	1257.077	23.5% (−1.2, 31.3)	0.139	3.4% (0, 11.8)	0.04
Model 4	1241.303	30.4% (0, 38.9)	0.04	10.4% (1.7, 21.5)	<0.01

Model 1: incorporating WC; model 2: variables in model 1 and FHD; model 3: variables in model 2 and HbA1c; model 4: variables in model 3 and FPG. AIC: Akaike's information criterion; CI: confidence interval; FHD: family history of diabetes; FPG: fasting plasma glucose; HbA1c: hemoglobin A1c; HR: hazard ratio; IDI: integrated discrimination improvement; NRI: net reclassification index; WC: waist circumference.

## Data Availability

The data used to support the findings of this study are available from the corresponding author upon request.

## Supplementary Materials

Supplementary Materials Table S1: univariate Cox proportional-hazards regression models for predicting prediabetes.

## References

[1] Nathan D. M., Davidson M. B., DeFronzo R. A. (2007). Impaired fasting glucose and impaired glucose tolerance: implications for care. *Diabetes Care*.

[2] Tabák A. G., Herder C., Rathmann W., Brunner E. J., Kivimäki M. (2012). Prediabetes: a high-risk state for diabetes development. *The Lancet*.

[3] Huang Y., Cai X., Mai W., Li M., Hu Y. (2016). Association between prediabetes and risk of cardiovascular disease and all cause mortality: systematic review and meta-analysis. *BMJ*.

[4] Crane P. K., Walker R., Hubbard R. A. (2013). Glucose levels and risk of dementia. *The New England Journal of Medicine*.

[5] Cho N. H., Shaw J. E., Karuranga S. (2018). IDF Diabetes Atlas: global estimates of diabetes prevalence for 2017 and projections for 2045. *Diabetes Research and Clinical Practice*.

[6] Li Y., Teng D., Shi X., Qin G., Qin Y., Quan H. (2020). Prevalence of diabetes recorded in mainland China using 2018 diagnostic criteria from the American Diabetes Association: National cross sectional study. *BMJ*.

[7] Pan X. R., Li G. W., Hu Y. H. (1997). Effects of diet and exercise in preventing NIDDM in people with impaired glucose tolerance: the Da Qing IGT and diabetes study. *Diabetes Care*.

[8] Vatcheva K. P., Fisher-Hoch S. P., Reininger B. M., McCormick J. B. (2020). Sex and age differences in prevalence and risk factors for prediabetes in Mexican-Americans. *Diabetes Research and Clinical Practice*.

[9] Aldossari K. K., Aldiab A., Al-Zahrani J. M. (2018). Prevalence of prediabetes, diabetes, and its associated risk factors among males in Saudi Arabia: a population-based survey. *Journal of Diabetes Research*.

[10] Tsimihodimos V., Gonzalez-Villalpando C., Meigs J. B., Ferrannini E. (2018). Hypertension and diabetes mellitus coprediction and time trajectories. *Hypertension*.

[11] Wen J., Wang A., Liu G. (2020). Elevated triglyceride-glucose (TyG) index predicts incidence of prediabetes: a prospective cohort study in China. *Lipids in Health and Disease*.

[12] Wu J., Gong L., Li Q. (2017). A novel visceral adiposity index for prediction of type 2 diabetes and pre-diabetes in Chinese adults: a 5-year prospective study. *Scientific Reports*.

[13] Ruchat S. M., Rankinen T., Weisnagel S. J. (2010). Improvements in glucose homeostasis in response to regular exercise are influenced by the PPARG Pro12Ala variant: results from the HERITAGE family study. *Diabetologia*.

[14] Shen X. M., Huang Y. Q., Zhang X. Y., Tong X. Q., Zheng P. F., Shu L. (2020). Association between dietary patterns and prediabetes risk in a middle-aged Chinese population. *Nutrition Journal*.

[15] Tricò D., Galderisi A., Mari A., Santoro N., Caprio S. (2019). One-hour post-load plasma glucose predicts progression to prediabetes in a multi-ethnic cohort of obese youths. *Diabetes, Obesity and Metabolism*.

[16] Wang H., Zheng X., Bai Z. H. (2020). A retrospective population study to develop a predictive model of prediabetes and incident type 2 diabetes mellitus from a hospital database in Japan between 2004 and 2015. *Medical Science Monitor*.

[17] Fujiati I. I., Damanik H. A., Bachtiar A., Nurdin A. A., Ward P. (2017). Development and validation of prediabetes risk score for predicting prediabetes among Indonesian adults in primary care: cross-sectional diagnostic study. *Interventional Medicine and Applied Science*.

[18] Xueyao Y., Saifei Z., Dan Y. (2014). Circulating fractalkine levels predict the development of the metabolic syndrome. *The Internet Journal of Endocrinology*.

[19] Tchernof A., Després J. P. (2013). Pathophysiology of human visceral obesity: an update. *Physiological Reviews*.

[20] Kahn S. E., Hull R. L., Utzschneider K. M. (2006). Mechanisms linking obesity to insulin resistance and type 2 diabetes. *Nature*.

[21] Wagner R., Thorand B., Osterhoff M. A., Müller G., Böhm A., Meisinger C. (2013). Family history of diabetes is associated with higher risk for prediabetes: a multicentre analysis from the German center for diabetes research. *Diabetologia*.

[22] Rodríguez-Moran M., Guerrero-Romero F., Aradillas-García C. (2010). Obesity and family history of diabetes as risk factors of impaired fasting glucose: implications for the early detection of prediabetes. *Pediatric Diabetes*.

[23] Watt G. P., Vatcheva K. P., Griffith D. M. (2016). The precarious health of young Mexican American men in South Texas, cameron county hispanic cohort, 2004–2015. *Preventing Chronic Disease*.

[24] Janghorbani M., Amini M. (2011). Normal fasting plasma glucose and risk of prediabetes and type 2 diabetes: the Isfahan diabetes prevention study. *The Review of Diabetic Studies*.

[25] Taheri F., Bijari B., Kazemi T., Namakin K., Zardast M., Chahkandi T. (2015). Prevalence of high normal FBS and prediabetes among adolescents in Birjand, East of Iran, 2012. *Journal of Education and Health Promotion*.

[26] Luo L., Li X., Gao Z. (2020). The value of waist circumference, waist height ratio and visceral adiposity index in predicting new prediabetes in women over 40 years old in Dalian. *Chinese Journal of Diabetes*.

[27] Chiu T. H. T., Huang H. Y., Chiu Y. F. (2014). Taiwanese vegetarians and omnivores: dietary composition, prevalence of diabetes and IFG. *PLoS One*.

[28] Krishnadath I. S. K., Nahar-van Venrooij L. M. N., Jaddoe V. W. V., Toelsie J. R. (2016). Ethnic differences in prediabetes and diabetes in the Suriname health study. *BMJ Open Diabetes Research & Care*.

[29] Okwechime I. O., Roberson S., Odoi A. (2015). Prevalence and predictors of pre-diabetes and diabetes among adults 18 years or older in Florida: a multinomial logistic modeling approach. *PLoS One*.

