# Supplementary material for: A Prediction Model for Prediabetes Risk in Middle-Aged and Elderly Populations: A Prospective Cohort Study in China
Source: Int J Endocrinol. 2021 Nov 11;2021:2520806. doi: 10.1155/2021/2520806 (PMC8601847; doi:10.1155/2021/2520806)
Supplement: Supplementary Materials — Table S1: univariate Cox proportional-hazards regression models for predicting prediabetes. [file 2520806.f1.docx]

**Supplementary data**

Table S1 Univariate cox proportional-hazards regression models for predicting prediabetes

| Characteristic | HR (95%CI) | p value |
| --- | --- | --- |
| Age, years | 1.023 (0.994, 1.052) | 0.120 |
| Gender | 1.432 (0.985, 2.082) | 0.060 |
| BMI, kg/m2 | 1.069 (0.999, 1.144) | 0.055 |
| WC, cm | 1.039 (1.018, 1.061) | <0.001 |
| FPG, mmol/L | 2.793 (1.912, 4.078) | <0.001 |
| FINS, uIU/mL | 1.045 (1.014, 1.076) | 0.005 |
| 2hPG, mmol/L | 1.344 (1.144, 1.580) | <0.001 |
| HbA1c, % | 2.117 (1.443, 3.106) | <0.001 |
| SUA, μmol/L | 1.002 (1.000, 1.004) | 0.045 |
| TG, mmol/L | 1.099 (0.986, 1.225) | 0.088 |
| HDL-C, mmol/L | 0.645 (0.380, 1.092) | 0.103 |
| Family history of diabetes, n (%) | 1.914 (1.313, 2.790) | 0.001 |
| Regular exercise, n (%) | 0.956 (0.655, 1.397) | 0.818 |
| Hypertension, n (%) | 1.353 (0.903-2.027) | 0.143 |
| Smoking, n (%) |  |  |
| Never | 1 |  |
| Former | 1.225 (0.781-1.919) | 0.377 |
| Current | 2.263 (1.253-4.088) | 0.007 |
| Alcohol drinking, n (%) |  |  |
| Never | 1 |  |
| Former | 1.364 (0.859-2.166) | 0.189 |
| Current | 1.287 (0.810-2.044) | 0.285 |
| Dietary patterns, n (%) |  |  |
| main vegetable dishes | 1 |  |
| balance meats and vegetables | 1.114 (0.504-2.462) | 0.789 |
| main meat dishes | 0.895 (0.573-1.397) | 0.625 |
| Nature of occupation, n (%) |  |  |
| manual work | 1 |  |
| physical and mental work | 0.988 (0.593, 1.647) | 0.963 |
| mental work | 1.296 (0.785, 2.138) | 0.310 |
| Education level, n (%) |  |  |
| High school or above | 1 |  |
| Middle school or below | 1.031 (0.709-1.499) | 0.874 |

Abbreviations: BMI, body mass index; WC, waist circumference; FPG, fasting plasma glucose; FINS, fasting serum insulin; HbA1c, hemoglobin A1c; HDL-C, high density lipoprotein-cholesterol; SUA, serum uric acid; TG, triglycerides; 2hPG, 2 h plasma glucose.
